# Iatrogenic nerve injury in primary and revision reverse total shoulder arthroplasty

**DOI:** 10.1007/s00402-022-04641-9

**Published:** 2022-10-17

**Authors:** Laura A. Hruby, I. Unterfrauner, F. Casari, P. Kriechling, S. Bouaicha, K. Wieser

**Affiliations:** 1 Department of Orthopaedics, Balgrist University Hospital, University of Zurich, Zurich, Switzerland; 2 Department of Orthopaedics and Trauma Surgery, Medical University of Vienna, Vienna, Austria

**Keywords:** Arthroplasty, replacement, shoulder, Peripheral nerve injuries, Iatrogenic disease, Intraoperative complications

## Abstract

**Introduction:**

Iatrogenic nerve injury in orthopedic surgery can impair functional outcomes. During the last years, a steady increase in the number of performed reverse total shoulder arthroplasties has been reported and complications associated with this procedure are continuously described. Neurological complications, however, remain underreported. The aims of this study were to calculate the incidence of iatrogenic nerve injury after primary and revision reverse total shoulder arthroplasty in a large patient cohort, as well as identify associated patient-and surgery-related risk factors.

**Materials and methods:**

A retrospective review of our institution’s internal Reverse Total Shoulder Arthroplasty (RTSA) database from September 2005 to December 2019 was undertaken and 34 patients with iatrogenic nerve injuries were identified, resulting in a neurological complication rate of 2.6%. Group comparisons between patients with nerve injuries (*n* = 34) and the remaining cohort without nerve injuries (*n* = 1275) were performed to identify patient- and surgery-related risk factors.

**Results:**

Of the 34 cases with iatrogenic nerve injury, damage to terminal nerve branches occurred in 21 patients, whereas a brachial plexus lesion was diagnosed in the other 13. Nerve revision surgery was necessary in four patients. At final follow-up 13 patients (45%) had residual motor deficits and 17 (59%) had residual sensory deficits. Higher numbers of previous surgeries of the affected shoulder correlated with subsequent nerve injury (*p* = 0.035). Operative time was significantly longer in patients, who developed a neurologic deficit, showing a correlation between duration of surgery and occurrence of nerve injury (*p* = 0.013). Patients with neurologic complications were significantly younger than patients without nerve damage (median 68 vs. 72 years, *p* = 0.017).

**Conclusions:**

In specialists’ hands reverse total shoulder arthroplasty is a rather safe procedure regarding the risk of neurologic injury. However, multiple previous surgeries of the affected shoulder increase the risk of neurological complications. Cases with post-operative neurologic compromise are rare and usually recover well, with few patients suffering long-term functional deficits from iatrogenic nerve injury.

**Level of evidence:**

Level III, retrospective cohort study.

## Introduction

Iatrogenic nerve injuries associated with shoulder surgery may lead to patient disability and distress, including long-lasting sensorimotor deficits, intractable neuropathic pain, increased costs, additional surgery and possible physician litigation [1, 2]. During the last decades, reverse total shoulder arthroplasty (RTSA) has gained increased popularity, not only due to an expansion of appropriate indications but also because of improvements in prosthesis design and implantation technique [3–5]. Modern western societies show a continuous rise in life expectancy and more recently RTSA is also increasingly performed in younger patients [3, 5, 6].

Previously published studies on risks and complications in RTSA surgery are numerous and diverse, with overall complication rates ranging from 5 to 70% [7–9]. In decreasing frequencies these include scapular notching; prosthesis instability and dislocation; glenoid component loosening and dislocation; infection; humeral complications and acromial fractures [10–12]. Neurological complications following reverse shoulder arthroplasty remain underreported. If at all, general neurovascular complications are presented in absolute and relative frequencies. Most studies lack further specification on the nerves injured [9], withhold information on the presumed mechanism of injury, do not present clinical signs of recovery in the post-operative period or include only limited patient numbers and short observation periods [13]. To the best of our knowledge this is the first report to critically review neurological complications following primary as well as revision RTSA in a large patient cohort over a 14-years period.

The aims of this study were to calculate the incidence of iatrogenic nerve injury after primary and revision RTSA in a specialized academic shoulder department, identify associated patient-and surgery-related risk factors and recognize possible inadvertencies during surgical handling.

## Materials and methods

The conduction of this study followed the ethical principles of the Helsinki Declaration and institutional review board approval was received from the Cantonal Ethics Committee Zurich (no. 2018-01494). The included patients gave written informed consent.

### Patients

A retrospective review of our institution’s internal Reverse Total Shoulder Arthroplasty (RTSA) database from September 2005 to December 2019 was undertaken to identify all patients with post-operative iatrogenic nerve injuries associated with the primary implantation of a reverse total shoulder prosthesis as well as with RTSA revision surgery. Thirty-four patients were included in this study after applying the following exclusion criteria:

- previous neurologic injury, which preceded implantation of the shoulder prosthesis
- post-operative nerve irritation/injury unrelated to the surgical procedure, such as radiculopathy or peripheral nerve compression as in carpal tunnel syndrome
- transient subjective sensory symptoms without physical findings and/or neurophysiological correlate
- tumor resection of the proximal humerus as the surgical indication for RTSA

Table 1 provides an overview of patient demographics and surgical characteristics.

**Table 1:** Patient demographics and surgical characteristics for patients with iatrogenic nerve injury after RTSA (*n* = 34)

Variables	Value
Sex (no., [%])	
Male	11 (32%)
Female	23 (68%)
Age (yr)^a^	68 (45–86)
Body mass index^a^ (kg/m^2^)	24.9 (19.7–36.5)
Previous surgery of the affected shoulder	
Yes	21 (62%)
No	13 (38%)
ASA score (no., [%])	
ASA 1	4 (12%)
ASA 2	24 (71%)
ASA 3	5 (15%)
ASA 4	1 (3%)
Primary implantation (no., [%])	
Yes	27 (79%)
Revision RTSA	7 (21%)
Indications for primary RTSA (no., [%])	
Cuff tear arthropathy	7 (21%)
Hamada IV^b^	4 (12%)
Hamada V^b^	3 (9%)
Irreparable cuff tear	8 (24%)
Hamada I^b^	4 (12%)
Hamada II^b^	4 (12%)
Primary osteoarthritis	3 (9%)
A2 glenoid^c^	1 (3%)
B1 glenoid^c^	1 (3%)
B3 glenoid^c^	1 (3%)
Previously failed ORIF	4 (12%)
Humeral head necrosis	1 (3%)
Proximal humeral fracture	3 (9%)
Persistent instability	1 (3%)
Revision RTSA (no., [%])	
Conversion from hemi-arthroplasty	4 (12%)
Conversion from anatomical TSA	2 (6%)
Component change of RTSA	1 (3%)
Surgical exposure (no., [%])	
Deltopectoral	32 (94%)
Superolateral	2 (6%)
Humeral stem^d^ (no., [%])	
Standard stem	27 (79%)
Fracture stem	6 (18%)
Long revision stem	1 (3%)
Stem fixation (no., [%])	
Cemented	16 (47%)
Press-fit	18 (53%)
Type of anesthesia (no., [%])	
Interscalene catheter	29 (85%)
General anesthesia	4 (11.8%)

*ASA* American Society of Anesthesiologists, *ORIF* open reduction and internal fixation, *RTSA* reverse total shoulder arthroplasty, *TSA* total shoulder arthroplasty, *yr* years

^a^The values are given as median (range in brackets)

^b^Classified according to the Hamada classification [14]

^c^Classified according to the modified Walch classification [15]

^d^All patients received the Zimmer Anatomical/Reverse system

Of the included patients, 23 were female (68%) and 11 were male (32%). The median age at surgery was 68 ± 10 years (range 45–86 years). The mean BMI was 25.9 ± 4.5 (range 19.7–36.5). Twenty-one patients (62%) had one or more (up to five) previous surgeries of the affected shoulder. The physical status as measured with the ASA (American Society of Anesthesiologists) classification was graded ASA 1 in four patients, ASA 2 in 24, ASA 3 in five and ASA 4 in one patient.

Primary implantations (*n* = 27) as well as revision surgeries (*n* = 7) were included, i.e., conversions from hemi-arthroplasty to RTSA, conversions from anatomical total shoulder prosthesis to RTSA and RTSA component changes. The indications for primary RTSA were variable: seven patients had a cuff tear arthropathy (classified according to Hamada [14]; grade V in three patients, grade IV in four patients) and eight had irreparable rotator cuff tears (Hamada grade I in four patients, and grade II in the other four); three suffered from primary glenohumeral arthritis (evaluation of axial CT images showed an A2 glenoid in one patient, B1 glenoid in one patient, and B3 glenoid in one patient according to the modified Walch classification [15]); four had previously failed open reduction and internal fixation (ORIF) surgery; one had a humeral head necrosis without preceding proximal humeral fracture; three patients had acute proximal humeral fractures ineligible for ORIF; and one had persistent shoulder instability refractory to preceding stabilization procedures. The seven revision surgeries included conversion from failed hemi-arthroplasty to RTSA (*n* = 4), conversion from anatomical TSA (*n* = 2) and change of prosthetic components after RTSA (*n* = 1).

The following data related to post-operative nerve injury were extracted from patient records: timepoint of discovery of sensory and/or motor deficits after surgery, type and (presumed) location of nerve injury, clinical evidence of functional deficits over time, electromyographic (EMG) and nerve conduction study (NCS) results, need of revision surgery, and finally, signs and progression of recovery at final follow-up. Strength in affected muscles was rated clinically using the BMRC grading scale. Sensory disturbances were documented as dysesthesia, paresthesia, and anesthesia/numbness. Surgical reports were screened for possible reasons for iatrogenic nerve injury. Types of anesthesia including nerve blocks and/or catheters were documented.

Mean follow-up in the study cohort was 61 ± 41 months (range 3–130 months). All 34 patients were included in the descriptive analysis. Five patients, who did not reach a minimum follow-up of 1 year, were excluded from the study leaving 29 patients for clinical outcome analysis. Of the five excluded patients, three had been lost to follow-up and two had been followed for less than 1 year.

### Surgical technique

A detailed description of the surgical procedure of RTSA implantation standardized at our institution can be found elsewhere [6]. Surgical characteristics in the study cohort are shown in Table 1.

In 32 of the 34 cases (94%) a deltopectoral exposure was used, while a superolateral delta-split approach was performed in the remaining two (6%). All patients received a Zimmer Anatomical/Reverse RTSA with a standard stem (27 patients), fracture stem (6 patients) or long revision stem (1 patient). In 16 patients (47%) the humeral component was cemented and press-fitted in the other 18 (53%). In 29 patients (85%) an interscalene catheter with ropivacaine (Sintetica, Switzerland) was installed preoperatively for patient-controlled analgesia and withdrawn 2 days postoperatively, 28 of these received general anesthesia with endotracheal intubation. In four patients (11.8%) general anesthesia without regional nerve blockade or interscalene catheter was applied (for specific anesthesiological data see Table 4). In none of the cases rigid arm fixations (such as Spider or Trimano arm holders) were used.

### Statistical analysis

All statistical analyses were performed using the software SPSS, version 26.0 (IBM, Armonk, NY, USA). To describe the study cohort absolute and relative frequencies were calculated. Group comparisons between the study cohort (patients with iatrogenic nerve injury) and the remaining cohort of the RTSA database (patients without iatrogenic nerve injury) were performed. The Kolmogorov–Smirnov test was applied to test for possible normal distribution of the data. Both groups were compared with *t* test or Wilcoxon rank sum test depending if normal distribution was given. Statistical analysis was performed on the following factors to identify potential patient-related risk factors for neurologic injury: age, sex, BMI, ASA score, operative time, and number of previous surgeries. For significant different variables between both groups, correlations were calculated with Spearman correlation coefficients. To analyse the influence of the placement of an interscalene catheter on the occurrence of iatrogenic nerve injuries, a Fisher’s exact test was performed. To identify surgical risk factors for iatrogenic nerve injury, subgroups were created according to three different indications for RTSA: previous failed ORIF, revision surgery and proximal humeral fracture. These scenarios were defined a priori, since they were assumed to potentially increase the risk of neurologic compromise following RTSA according to clinical experience and available literature. Relative risk (RR) ratios to sustain nerve injuries were calculated for those three different surgical indications. To document differences in the surgical experience in both groups, surgeons were grouped according to their educational level: resident, attending, consultant/leading physician of shoulder department, chief surgeon. To identify the influence of the surgeon’s experience on the occurrence of iatrogenic nerve injury, a point-biserial correlation was calculated.

A *p* value ≤ 0.05 (two-tailed) was considered significant. Correlation coefficients > 0.3 were regarded moderate and coefficients over > 0.6 strong, respectively.

## Results

**Fig. 1:**
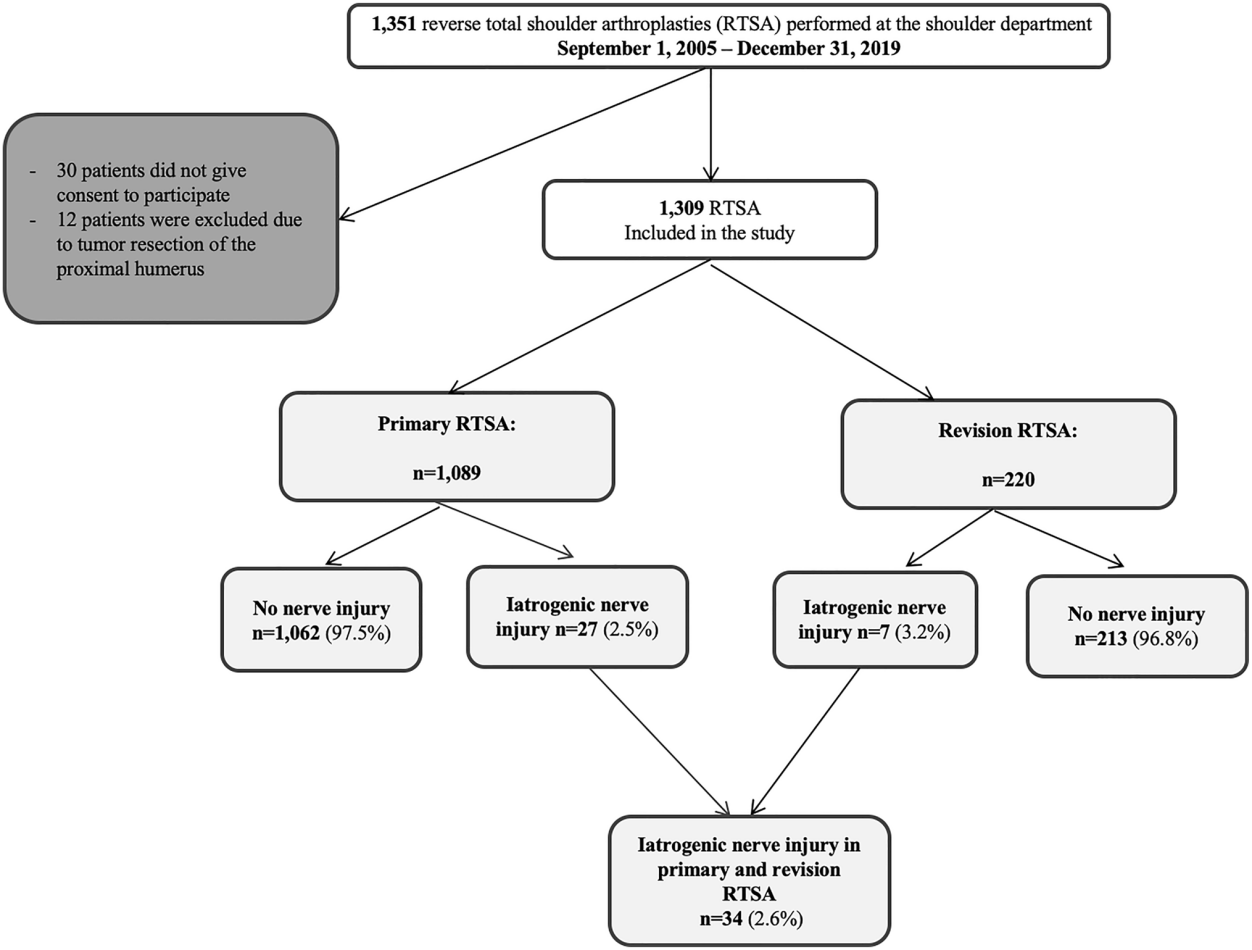
Flowchart showing the composition of the study cohort (*n* = 34)

Between September 2005 to December 2019, 1351 reverse total shoulder arthroplasties were performed at our institution. 1321 patients gave written informed consent to have their data reviewed and published in a scientific endeavor. Twelve arthroplasties were performed due to resection of a tumor of the proximal humerus and were excluded from the analysis, leaving a total number of 1309 RTSAs (see Fig. 1). In 34 cases (= 2.6%), iatrogenic nerve injury had occurred.

### Characterization of nerve injury

Damage to major terminal nerves occurred in 21 patients (62%), whereas a lesion at the brachial plexus either at trunk or cord level was diagnosed in the other 13 (38%) (see Table 2).

**Table 2:** Characterization of nerve injury (*n* = 34)

	No. of cases (%)
Axillary nerve	10 (29%)
Isolated	7 (21%)
Combined with SCN	2 (6%)
Combined with ulnar nerve	1 (3%)
Suprascapular nerve	3 (9%)
Isolated	1 (3%)
Combined with axillary nerve	2 (6%)
Ulnar nerve	5 (15%)
Isolated	3 (9%)
Combined with radial nerve	1 (3%)
Combined with LACB	1 (3%)
Radial nerve	4 (12%)
Isolated	3 (9%)
Combined with ulnar nerve	1 (3%)
Median nerve	2 (6%)
Plexus lesion	13 (38%)
Multiple levels	5 (15%)
Upper plexus lesion	5 (15%)
Lower plexus lesion	3 (9%)

Descriptive characterization of nerve injuries within the study cohort

*LACB* lateral antebrachial cutaneous branch

Isolated sensory deficits were present in four patients (12%), sole motor dysfunction was recognized in five patients (15%) and mixed sensorimotor deficits were seen in the remaining 25 (74%).

Isolated axillary nerve palsy was present in seven patients and combined with injuries to other terminal nerves in another three cases. The axillary nerve as a terminal nerve was therefore affected in 29% of all patients. Deltoid muscle strength was additionally compromised in nine patients with plexus lesions, therefore, resulting in deltoid paresis (with various degrees) in overall 19 patients (56%). Three patients (9%) had lower plexus lesions, where hand function and sensation were impaired. Radial nerve function was compromised in four patients (12%), ulnar nerve function in five (15%) and median nerve function in two patients (6%), respectively. The suprascapular nerve was injured in three patients (9%).

### Severity and treatment of iatrogenic nerve injury

In 4 of 34 cases (12%) nerve revision or reconstructive surgery was necessary.

Of these, neurotmesis, i.e., cross-sectional nerve transection, was apparent during surgery in two patients. In one case, the main trunk of the axillary nerve was unintentionally transected with the scalpel and reconstructed with a sural nerve graft, which recovered to deltoid strength M3 with complete anesthesia in the lateral upper arm at final follow-up. In the other case, a small branch of the anterior axillary nerve was transected and sutured end-to-end micro-surgically without an interposition nerve graft. Full motor and sensory recovery ensued.

In the remaining two patients axonotmesis of the radial nerve had occurred. In one patient a humeral stem fracture during primary RTSA required cerclage wiring with subsequent drop hand and hypesthesia at the dorsolateral forearm and dorsal thumb. Revision surgery after 6 weeks revealed mechanical damage of single fascicles of the radial nerve at the fracture site as the mechanism of injury. Neurolysis was performed to treat this axonotmetic injury. At final follow-up wrist and finger extensors showed M4 strength and sensation in the innervation area of the superficial branch of the radial nerve was improved but still partially impaired. In the other patient the same mechanism of injury had occurred, i.e., partial mechanical damage of the radial nerve at humeral fracture site upon stem breakage and cerclage wiring. This patient received secondary reconstructive surgery to improve motor function (Merle d’Aubigné reconstruction) externally and was lost to follow-up.

In the other 88% of patients, where less severe clinical deficits ensued post-operatively and neurapraxic and axonotmesis injuries were suspected upon neurophysiological assessment, no revision surgery was performed and patients received physiotherapy and—in some cases—surface electrical stimulation as the conservative treatment.

### Recovery of nerve function

Of the 29 patients, who reached a follow-up of more than 1 year, full motor function without any sign of muscle weakness was present in 16 patients (55%) at final follow-up. Thirteen patients (45%) showed residual motor deficits in the affected limb. In seventeen patients (59%) sensory deficits were persistent, while in 12 patients (41%) intact sensory function was present.

Of the 19 patients (56%) with deltoid muscle affection good deltoid recovery (M4-5) ensued in 13 patients, who showed a median global shoulder flexion of 120° (range 70°–170°) and shoulder abduction of 130° (range 45°–160°). Four patients with weak deltoid recovery (M2–3) showed a median global shoulder flexion of 30° (range 15°–40°) and shoulder abduction of 38° (range 15°–40°). Two were lost to follow up.

### Patient- and surgery-related risk factors

Regarding potential risk factors for iatrogenic nerve injury, patient BMI, history of tobacco use, and patient sex were not found to correlate with the occurrence of nerve injury (Table 3). Patients with and without nerve damage did not differ in regards to general physical status as measured with the ASA score (*p* = 0.170). Although there was a strong trend, previous surgery of the affected shoulder did not significantly increase the risk for iatrogenic nerve injury per se (RR 1.23, 95% CI 1.02–1.75; *p* = 0.074). There was, however, a significant difference in the number of previous surgeries (*p* = 0.035). Operative time was found to be statistically significant in the development of a neurologic deficit, showing a weak correlation between duration of surgery and occurrence of nerve injury (*r* = 0.07, *p* = 0.013); those who had an iatrogenic nerve injury had a longer operative time (median 190 min) compared with those with normal postoperative nerve function (median 167 min). Patients with neurologic complications were significantly younger than patients without nerve damage (median 68 vs. 72 years, *p* = 0.017). The remaining parameters in crude group comparisons were not significant: sex (*p* = 0.378), BMI (*p* = 0.059) and operation side (*p* = 0.683) (see Table 3).

**Table 3:** Potential risk factors for iatrogenic nerve injury

Covariate	Overall (*n* = 1309)	Stratified by iatrogenic nerve injury		*p* value*
		Yes = 34	No = 1275	
Sex				*p* = 0.378
Female	790 (60.4%)	23 (67.6%)	767 (60.2%)	
Male	519 (39.6%)	11 (32.4%)	508 (39.8%)	
Age^a^				***p* = 0.017**
Median (range), years	72 (24–94)	68 (45–86)	72 (24–94)	
BMI^a^				*p* = 0.059
Median (range), kg/m^2^	26.7 (14.2–44.2)	24.9 (19.7–36.5)	26.7 (14.2–44.2)	
Tobacco use				*p* = 0.284
Yes	190 (15.2%)	7 (21.9%)	183 (15.0%)	
No	1,063 (84.8%)	25 (78.1%)	1,083 (85.0%)	
Missing	56 (4.3%)			
Operative time^a^				***p* = 0.013**
Median (range), min	170 (35–450)	190 (98–430)	167 (35–450)	
Previous surgeries^a^				***p *= 0.035**
Median numbers of surgeries (range)	0 (0–12)	1 (0–5)	0 (0–12)	

A history of tobacco use, sex, and BMI were not found to correlate with the occurrence of iatrogenic nerve injury. Age at the time of surgery was significantly lower in the nerve injury cohort, while operative time was significantly longer in the study cohort. A higher number of previous surgeries was found a statistically significant risk factor to sustain iatrogenic nerve injury

*BMI* body mass index

^a^The values are given as median (range in brackets). Categoric variables are presented as numbers (percentage in brackets)

^*^p values were calculated by either the *χ*^2^ or Fisher exact test for categorical variables, or the Wilcoxon rank sum test for non-normally distributed numerical variables

A *p* value ≤ 0.05 (two-tailed) was considered significant in bold

Table 4 shows absolute and relative frequencies of applied anesthesia for both groups. The placement of an interscalene catheter with ropivacaine had no influence on the occurrence of iatrogenic nerve injuries (*p* = 0.374).

**Table 4:** Anesthesiological data

	No iatrogenic nerve injury *n* = 1275	Iatrogenic nerve injury *n* = 34
Form of anesthesia^a^		
General anesthesia with endotracheal intubation	940 (73.7%)	32 (94.1%)
With ISC	737 (57.8%)	28 (82.4%)
Without ISC	188 (14.7%)	4 (11.8%)
Unknown	15 (1.2%)	0
Exclusive regional anesthesia	265 (20.8%)	1 (2.9%)
With ISC	197 (15.5%)	1 (2.9%)
Without ISC	43 (3.4%)	0
Unknown	25 (1.9%)	0

*ISC* interscalene cathete with ropivacaine (Sintetica, Switzerland)

^a^Data of applied anasthesia are missing in 70 cases in the group without iatrogenic nerve injury and in 1 case with iatrogenic nerve injury

Regarding potentical surgery-specific risk factors, prosthetic revision surgery (RR 1.23, 95% CI 0.63–2.41; *p* = 0.55), previous failed ORIF (RR 1.56, 95% CI 0.61–4.00; *p* = 0.36) and proximal humeral fracture as the surgical indication for RTSA (RR 1.46; 95% CI 0.49–4.40; *p* = 0.50) were not identified as risk factors for iatrogenic nerve injury.

The surgeon’s experience according to the educational level (resident, attending, consultant/leading physician of shoulder department, chief surgeon) had no influence on the occurrence of nerve injury (*p* = 0.128). For detailed data see Table 5.

**Table 5:** Surgeon’s experience according to educational level

	No iatrogenic nerve injury *n* = 1275	Iatrogenic nerve injury *n* = 34
Educational level^a^		
Resident	6 (0.5%)	0
Attending	253 (19.8%)	6 (17.6%)
Consultant/leading physician of shoulder department	447 (35.1%)	7 (20.6%)
Chief surgeon	563 (44.2%)	21 (61.8%)

^a^Data of educational level are missing in 6 cases in the group without iatrogenic nerve injury

## Discussion

To our knowledge, this is the largest investigation of iatrogenic nerve injuries in primary as well as revision reverse total shoulder arthroplasty in the literature. In this large-scale study including an observation period of fourteen years with 1309 consecutive RTSAs, we could confirm a low rate of iatrogenic nerve injury of 2.6%. This ratio is in line with previously published data, which included lower patient numbers [16, 17]. Although Kim and colleagues [18] reported of a much higher prevalence of iatrogenic nerve injuries (34 of 182 consecutive RTSAs, i.e. 19%), the distribution and characterization of nerve injuries (see Table 2) in our cohort was found to be very similar.

In 19 of 34 patients (56%) axillary nerve dysfunction and consequent deltoid weakness was present after surgery. Neurotmesis of the axillary nerve was evident in two cases and was recognized intra-operatively necessitating nerve reconstruction with a sural nerve graft and direct end-to-end suture, respectively. Suprascapular nerve palsy was evident in three patients (9%) post-operatively.

In a prospective EMG analysis Lopiz and colleagues [19] revealed a high prevalence of axillary and suprascapular nerve injuries pre- and post-operatively after RTSA in patients with cuff tear arthropathy. Pre-operative alterations were either chronic or disuse injuries due to long-standing pain and an insufficient rotator cuff. Acute iatrogenic injury to the axillary nerve was evident in EMG analysis in 31.5% of patients, the same relative frequency was documented for injuries to the suprascapular nerve. Lädermann and colleagues [13] reported similar results with pathological EMG alterations of the deltoid seen in 47% after RTSA. In this study, EMG measurements were performed post-operatively regardless of the presence of neurological deficits. At 6 month follow-up none, however, had motor deficits and in the EMG follow-up measurements eight of nine deltoid lesions had recovered completely. The value of electromyography in subclinical neurological affections, therefore, seems controversial, as signs of acute denervation do not seem to inflict on the final functional outcome. In a cadaveric study by Lädermann and colleagues [20], the authors described the close proximity of the axillary nerve to the posterior metaphysis and the humeral prosthetic component of a reverse shoulder prosthesis putting it at risk of injury in this area. In an anatomical study by Leschinger et al. [21], the authors showed an increased risk of suprascapular nerve injury upon drilling and extraosseous screw placement during glenoid baseplate implantation in RTSA.

In our study cohort, brachial plexus affection with mixed sensorimotor deficits had been identified in 13 patients (38%, see Table 2). Increased strain on the brachial plexus, i.e. stretching and elongation, during reverse shoulder arthroplasty surgery has been shown in intra-operative nerve monitoring studies [22–24]. Furthermore, the desired lengthening of the involved arm after RTSA, has also been associated with a higher prevalence of nerve complications compared with anatomic shoulder arthroplasties (TSA) [13]. The presumed injury mechanism again is traction on the brachial plexus and its terminal nerve branches. Kim confirmed these earlier results, showing that arm lengthening of more than two centimeters following RTSA was associated with higher risks of neurological complications [18].

The risk of peripheral nerve injury due to regional anesthesia ranges from 0 to 5% [1, 25]. Possible injury mechanisms include neurotoxicity of the applied anesthetic, direct puncture of a nerval structure and compressive haematoma [1, 26–28]. In our study, we could rule out an influence of the placement of an interscalene catheter with ropivacaine on the occurrence of iatrogenic nerve injury in RTSA.

In six patients, ulnar neuropathy was present after RTSA. Since nerve conduction examinations revealed a nerve conduction block within the cubital tunnel in most patients, we had attributed these injuries to non-conforming positioning and bedding of the elbow during surgery or during the early post-operative course with increased compression and/or traction on the ulnar nerve [1].

To identify surgery-related risk factors we determined a priori three scenarios, which from a clinical point of view as well as considering the available literature had been assumed to potentially increase the probability of neurologic compromise following RTSA. These were revision surgery, proximal humeral fracture as the indication for a fracture prosthesis, and failed plate osteosynthesis. Although recent studies had shown a three-fold increased complication rate in revision RTSA surgery [11, 29], this was not the case for risk of iatrogenic nerve injury in our study cohort. Revision RTSA surgery could be rejected as a surgery-related risk factor for intra-operative nerve damage. Previous surgeries of the affected shoulder, however, were identified as a risk factor and there was a correlation between the number of previous surgeries and occurrence of nerve injury. This finding should prompt treating surgeons to consider careful surgical manipulation upon excision of extensive scar tissue [30]. On the other hand, proximal humeral fractures ineligible for open reduction and internal fixation as the surgical indication for RTSA did not increase the risk for iatrogenic nerve injury. This finding had been reported by others, showing no additional risk for complications at 1 year postoperatively compared with nonfracture patients [9]. RTSA for failed ORIF of proximal humeral fractures [31] did also not increase the risk of obtaining iatrogenic nerve injury in our study cohort.

In regards to patient-related risk factors, neither BMI nor the presence of comorbidities as measured with the ASA classification differed between patients with and without iatrogenic nerve injury. Nerve injury patients were significantly younger compared to patients without neurological compromise. We have no clear explanation for this interesting finding. Median operative time was significantly longer in patients who sustained an iatrogenic nerve injury, possibly indicating complicated conditions in terms of extensive scar tissue in cases of higher numbers of previous surgeries. The median operative time for both groups appeared to be comparatively long for a specialized center. This finding may be explained by wo main reasons: first, we have included an observation period of fourteen years dating back to 2005, where surgical experience with RTSA had not been as extended as of today. Secondly, this report included not only primary but also revision RTSAs, which naturally prolongs operative time.

While careful surgical handling, such as cautious placement and manipulation of retractors, as well as raising critical awareness also in assisting surgeons may be easily achieved and further improved, the inherent increase in humeral length and subsequent strain on the brachial plexus and its terminal nerves cannot be avoided in RTSA. Iatrogenic nerve injury during surgery may, therefore, be positively influenced with increasing experience; however, nerve complications due to the nonanatomic design and consequent biological as well as mechanical adaptions of RTSA with lengthening of the arm and elongation of the brachial plexus might remain a problem of this procedure [18, 23, 32].

### Study strengths and limitations

The presented study has several strenghts. After careful review of the literature, it represents the largest cohort series of primary and revision reverse total shoulder arthroplasty investigating post-operative neurologic compromise. The main interest of this study was to characterize iatrogenic nerve injuries sustained during reverse total shoulder arthroplasty including potential injury mechanisms. Secondly, we provide clinicians with a solid reference to stand on when counseling patients preoperatively regarding the risk of sensorimotor deficits following reverse total shoulder arthroplasty.

This study, however, also has several limitations including its retrospective nature.

Upon clinical suspicion of iatrogenic nerve injury following RTSA, patients were referred to our neurology department for a comprehensive neurological and neurophysiological examination. The parameters that were assessed included motor and sensory neurographies, nerve conduction velocity, and standard needle electromyographic assessment where applicable. Follow-up neurophysiological assessments were adapted to the progression of recovery. As such neurophysiological examinations were not standardized and varied greatly inter-individually depending on the suspected nerve injured, the presumed location and type of injury as well as technical possibilities of needle EMG. Since the obtained data could not be displayed following a specific structure, we decided not to include it in this study.

Although a variety of patient- and surgery-related risk factors were identified in the nerve injury cohort (*n* = 34) as compared to 1275 patients without neurological compromise, certain parameters were not assessed routinely in our RTSA database. Excessive lengthening of the arm, which has been shown to produce strain on the brachial plexus, was not routinely assessed and therefore was not included in our analysis.

## Conclusions

In this large cohort study of primary and revision reverse total shoulder arthroplasty, the total iatrogenic nerve injury complication rate was 2.6%. In specialists’ hands reverse total shoulder arthroplasty is a rather safe procedure regarding the risk of neurologic injury. However, multiple previous surgeries of the affected shoulder increase the likelihood of neurological complications. Cases with post-operative neurologic compromise usually recover well, with few patients suffering long-term functional deficits from iatrogenic nerve injury. Critical examination of peripheral sensorimotor function upon follow-up consultation and high index of suspicion for iatrogenic complications are warranted to allow early diagnosis and appropriate treatment if needed.
